# Investigation of swallowing function and swallowing-related quality of life after partial laryngectomy in Chinese patients with laryngeal carcinoma

**DOI:** 10.1186/s12955-019-1199-5

**Published:** 2019-07-26

**Authors:** Hui Yang, Dongfang Han, Xiaoyong Ren, Huanan Luo, Xiaomei Li

**Affiliations:** 1 Department of Otorhinolaryngology Head and Neck Surgery, the Second Affiliated Hospital of Xi’an Jiaotong University, Xi’an, Shaanxi China; 2 Department of Gynaecology and Obstetrics, the First Affiliated Hospital of Xi’an Jiaotong University, Xi’an, Shaanxi China; 3 School of Nursing, Health Science Center, Xi’an Jiaotong University, Xi’an, Shaanxi China

**Keywords:** Laryngeal carcinoma, Laryngectomy, Swallowing function, Quality of life

## Abstract

**Background:**

Swallowing function and swallowing-related quality of life (QoL) can be adversely affected in patients after partial laryngectomy, but are often neglected by patients and clinical workers. This study aimed to investigate the degree of swallowing function and swallowing-related QoL after partial laryngectomy in patients with laryngeal carcinoma.

**Methods:**

Sixty-eight hospitalized patients undergoing partial laryngectomy due to laryngeal carcinoma at the Second Affiliated Hospital of Xi’an Jiao Tong University were included in this prospective study. A general information questionnaire was used to collect baseline characteristics. The water swallow test and swallowing quality of life questionnaire (SWAL-QOL) were carried out the day before surgery and at 2, 4, 12, 24 and 48 weeks after surgery.

**Results:**

Swallowing dysfunction occurred in 1 case (1.5%) the day before surgery and in 49 (72.1%), 44 (64.7%), 33 (49.3%), 19 (28.4%) and 8 (11.9%) cases at 2, 4, 12, 24 and 48 weeks after surgery, respectively. Mean SWAL-QOL total scores were 4266.3 ± 232.0 the day before surgery, and 1992.9 ± 1062.4, 2473.9 ± 962.9, 3169.2 ± 753.6, 3696.7 ± 718.3 and 3910.8 ± 1510.4 at 2, 4, 12, 24 and 48 weeks, respectively. SWAL-QOL total scores increased gradually after operation, and the differences were statistically significant (*P* < 0.05). There was no statistical difference between postoperative 24 and 48 weeks (*P* = 0.379).

**Conclusions:**

Partial laryngectomy affects swallowing function and swallowing-related QoL in patients with laryngeal carcinoma. While swallowing function and swallowing-related QoL increase gradually over time, in some patients, nearly a year after surgery they are not fully restored. Therefore, attention should be paid during postoperative nursing to improve swallowing function.

## Background

Laryngeal cancer is one of the most common malignant tumors encountered in the otorhinolaryngology department [1]. In China it accounts for 1–5% of all malignant tumors, and for 11–22% of head and neck malignant tumors, and the incidence rate is still increasing [2, 3]. While early stage cancers are often treated by chemoradiotherapy, surgery is the most common treatment for advanced laryngeal cancer [4], including total and partial laryngectomies [5]. Total laryngectomy has a severe impact on the quality of life (QoL) of the patients due to the long term need for a tube and loss of speech [2, 6, 7]. Therefore, methods preserving the larynx in partial laryngectomy currently have extensive clinical use [8] and are recommended by clinicians [9].

The purpose of partial laryngectomy for laryngeal cancer patients is to preserve and reconstruct laryngeal function while ensuring the safety margin. Considering the tumor location, size, shape, range, pattern of growth and differentiation extent, the resection area can be expanded or reduced [10], using methods such as epiglottidectomy, and horizontal and vertical partial laryngectomies [11]. Regardless of type, partial laryngectomy will destroy and damage some of the local anatomical structure and nerve or muscle function of the larynx, thus causing different degrees of swallowing dysfunction [12, 13], which is a high-occurrence complication of partial laryngectomy [14].

It takes a long time for most patients after partial laryngectomy to recover the swallowing function as they rely on self-exercise or natural rehabilitation of nerve and muscle function [15]. In addition, these patients usually undergo standardized medical or nursing interventions, with few well-designed studies assessing swallowing function and specific QoL after partial laryngectomy [14, 16–19]. It was suggested that the patient-to-patient communication model could help solve swallowing problems in supracricoid partial laryngectomy (SCL) patients [17], with partial laryngectomy improving swallowing function postoperatively [18]. For further exploration, we specifically assessed swallowing-related QoL in Chinese patients with laryngeal carcinoma after partial laryngectomy in this study.

## Methods

### Patients

Consecutively laryngeal carcinoma patients undergoing partial laryngectomy in the department of otorhinolaryngology surgery, the Second Affiliated Hospital of Xi’an Jiao Tong University, were enrolled between January 2015 and December 2016 in this prospective study. Laryngeal carcinoma was staged according to the seventh edition of TNM staging of the American Joint Committee on Cancer (AJCC). Selection of partial laryngectomy was mainly based on the location, size and local invasion of the tumor. The inclusion criteria were: (1) postoperative histopathological diagnosis as laryngeal squamous cell carcinoma; (2) planned first partial laryngectomy; (3) no operation history for the head or neck within three months; (4) no existing or potential disease causing swallowing dysfunction; (5) no severe surgical complications; (6) consent to participate in this study. The exclusion criteria were: (1) abnormal communication due to consciousness disturbance; (2) recurrent tumor or partial laryngectomy performed several times. The study was carried out in accordance with the declaration of Helsinki and approved by the ethics committee of the Second Affiliated Hospital of Xi’an Jiao Tong University. Informed consent was obtained from all patients.

### Investigation tools

In this study, swallowing function was assessed by the water swallow test. A general information questionnaire along with the swallowing-related QoL questionnaire were used to investigate swallowing-related QoL. The water swallow test [20] is an experimental method proposed by Kubota Toshio for the evaluation of dysphagia, with clear classification and simple operation. The examination procedure of dysphagia was: the patient sat down to drink 30 mL of warm water; drinking time and cough status were observed. The following grading system was used: Grade 1, swallowing of water once successfully; Grade 2, swallowing of water over two or more attempts with no cough; Grade 3, swallowing of water once but with cough; Grade 4, swallowing of water in two or more attempts with cough; Grade 5, inability to swallow all the water and frequent cough. Grades 3–5 indicated dysphagia while Grades 1–2 reflected normal swallowing function.

A general information questionnaire was designed by the authors with reference to the relevant literature combined with clinical data of laryngeal carcinoma patients, including age, gender, ethnic, education level, marital status, occupation status, smoking and drinking statuses, condition of laryngeal diseases and operation selection. The Swallow Quality-of-Life Questionnaire (SWAL-QOL, Hong Kong, Chinese version) [21], with good reliability and validity in clinical trials [22] and evaluating the QoL in terms of swallowing-related factors, was introduced and translated by Lam & Lai into traditional Chinese [21]. The questionnaire contains 13 domains, of which 8 domains are related to swallowing, one domain comprises swallowing symptoms and frequencies, and four are common domains. The questionnaire includes 48 items totally, with 47 having 5-grade score options from 0, 25, 50, 75 to 100, which corresponded to swallowing-related QoL. The remaining item has a 2-grade score options from 0 to 100. The total score ranges from 0 to 4800; the higher the score, the better the swallowing-related QoL.

### Investigation methods

Medical records and preoperative discussions were consulted, and the patients were included according to the above inclusion and exclusion criteria. The general information questionnaire was completed after the patient provided a signed informed consent. Swallowing function was assessed by a trained investigator using the water swallow test. Then, SWAL-QOL was completed during outpatient follow-up the day before surgery and at 2, 4, 12, 24 and 48 weeks after operation. All the patients accepted unified guidance to fill out the questionnaire by themselves. The questionnaire was completed in about 10 min. The investigator checked the questionnaire to make sure that there were no missing items, and answers were not repeated several times in a random way; the collected data were valid.

### Statistical analysis

SPSS 17.0 (IBM, Armonk, NY, USA) was used to analyze the data obtained from the survey with the EPIDATA software (www.EpiData.dk). Continuous variables were expressed as mean ± standard deviation (SD), and repeated measures analysis of variance was used to compare differences in SWAL-QOL at different times. Categorical variables were expressed as frequency and percentage, and differences in swallowing function at different times were compared by the Chi-square test. *P* < 0.05 was considered statistical significant.

## Results

In this survey, a total of 68 general information questionnaires were issued and completed. In all, 408 SWAL-QOL questionnaires (6 for each patient) were issued, but one patient died after completing only three questionnaires, so a total of 405 effective questionnaires were collected, for a recovery rate of valid questionnaires of 99.3%.

Patient characteristics are shown in Table 1. The majority of patients were male (94.1%) and of Han ethnicity (95.6%), with a mean age of 61.9 ± 9.3 years.

**Table 1:** General information of the patients

Variable	Patients (*n* = 68)
Gender, n (%)	
Male	64 (94.1)
Female	4 (5.9)
Age (years), mean ± SD	61.9 ± 9.3
Ethnicity, n (%)	
Han	65 (95.6)
Others	3 (4.4)
Education level, n (%)	
Illiteracy	11 (16.2)
Primary school or junior high school	33 (48.5)
High school or secondary specialized school	16 (23.5)
University or above	8 (11.8)
Marital status, n (%)	
Married	62 (91.2)
Divorced / widowed	6 (8.8)
Occupation status, n (%)	
On-the-job	6 (8.8)
Retired	34 (50.0)
Others	28 (41.2)
Smoking, n (%)	
Never	19 (27.9)
Current smoker	40 (58.8)
Former smoker	9 (13.2)
Drinking, n (%)	
Never	38 (55.9)
Current drinker	23 (33.8)
Former drinker	7 (10.3)

Cancer stage distribution is shown in Table 2. The majority of patients were classified as tumor stage II (66.2%). Vertical partial laryngectomy was the most common choice of operation.

**Table 2:** Clinical conditions of the patients

Variable	Patients (*n* = 68)
Tumor stage, n (%)	
I	13 (19.1)
II	45 (66.2)
III	8 (11.8)
IV	2 (2.9)
T-stage, n (%)	
T1	44 (64.7)
T2	14 (20.6)
T3/T4	10 (14.7)
M-stage, n (%)	
M0	65 (95.6)
M1	3 (4.4)
N-stage, n (%)	
N0	54 (79.4)
N1/N2	14 (20.6)
Surgery type, n (%)	
Vertical partial laryngectomy	45 (66.2)
Horizontal partial laryngectomy	18 (26.5)
Horizontal and vertical partial laryngectomy	5 (7.4)
Lymph node dissection, n (%)	
Unilateral	25 (36.8)
Bilateral	12 (17.6)
No	31 (45.6)
Pathological type, n (%)	
Squamous cell carcinoma	68 (100)
Preoperatively induced chemotherapy, n (%)	
Yes	6 (8.8)
No	62 (91.2)
Postoperative radiotherapy, n (%)	
Yes	61 (89.7)
No	7 (10.3)

Swallowing function was adversely affected by surgery in 49 patients (72.1%) 2 weeks later (Table 3). Swallowing function slowly recovered over 48 weeks, and there was a significant improvement at 12 (*P* = 0.047), 24 (*P* = 0.022) and 48 (*P* = 0.021) weeks, but eight (11.9%) patients were still affected after 48 weeks.

**Table 3:** Swallowing function at different time points after partial laryngectomy

Time	N	Dysphagia (Grade 3–5), n (%)	Normal swallowing (Grade 1–2), n (%)
Preoperative 1 d	68	1 (1.5)	67 (98.5)
Postoperative 2 weeks	68	49 (72.1)	19 (27.9)
Postoperative 4 weeks	68	44 (64.7)	24 (35.3)
Postoperative 12 weeks	67	33 (49.3)	34 (50.7)
Postoperative 24 weeks	67	19 (28.4)	48 (71.6)
Postoperative 48 weeks	67	8 (11.9)	59 (88.1)

*P* < 0.001, postoperative 2 weeks vs. preoperative 1 d

*P* = 0.356, postoperative 4 weeks vs. postoperative 2 weeks

*P* = 0.047, postoperative 12 weeks vs. postoperative 4 weeks

*P* = 0.022, postoperative 24 weeks vs. postoperative 12 weeks

*P* = 0.021, postoperative 48 weeks vs. postoperative 24 weeks

SWAL-QOL total scores were decreased after surgery, but slowly recovered over the 48-week follow-up, as shown in Table 4. There were significant improvements in SWAL-QOL total score at all time points (*P* < 0.001) except the final one, since total scores were similar at 24 and 48 weeks (*P* = 0.379).

**Table 4:** SWAL-QOL scores at different time points after partial laryngectomy

Domain	Item	Preoperative 1 d (*n* = 68)	Postoperative 2 weeks (*n* = 68)	Postoperative 4 weeks (*n* = 68)	Postoperative 12 weeks (*n* = 67)	Postoperative 24 weeks (*n* = 67)	Postoperative 48 weeks (*n* = 67)
Burden	2	200.0 ± 0.0	69.5 ± 65.9^a^	82.7 ± 65.0^b^	122.8 ± 53.0^c^	155.5 ± 46.1^d^	182.6 ± 69.8
Eating duration and desire	5	436.7 ± 59.1	229.8 ± 145.6	276.1 ± 136.2^b^	352.9 ± 105.1^c^	413.6 ± 91.3^d^	400.4 ± 167.2
Symptom frequency	14	1210.1 ± 116.4	662.5 ± 343.9^a^	791.5 ± 298.2^b^	989.0 ± 231.3^c^	1115.1 ± 221.6^d^	1093.0 ± 453.0
Food selection	2	167.3 ± 31.2	56.6 ± 52.5^a^	82.7 ± 55.5^b^	119.1 ± 45.1^c^	149.6 ± 39.9^d^	151.1 ± 68.5
Communication	2	176.5 ± 25.1	72.1 ± 51.6^a^	96.0 ± 52.5^b^	125.7 ± 46.5^c^	148.9 ± 38.7^d^	151.8 ± 67.2
Fear	4	400.0 ± 0.0	166.9 ± 105.8^a^	214.3 ± 102.0^b^	276.5 ± 82.6^c^	327.9 ± 72.6^d^	322.1 ± 135.4
Mental health	5	500.0 ± 0.0	230.2 ± 170.9^a^	262.5 ± 134.4^b^	361.0 ± 109.2^c^	418.0 ± 93.5^d^	398.2 ± 171.0
Social	5	500.0 ± 0.0	171.0 ± 144.2^a^	222.4 ± 134.6^b^	296.3 ± 113.8^c^	367.7 ± 108.4^d^	363.6 ± 169.6
Fatigue and sleep	5	343.0 ± 72.4	202.6 ± 98.7^a^	269.1 ± 109.5^b^	334.9 ± 71.4^c^	390.8 ± 86.1^d^	380.9 ± 162.5
Indwelling gastric tube	1						
Yes (score = 0)		0	7 (10.3)^a^	6 (8.80)	3 (4.5)	2 (3.0)	2 (3.0)
No (score = 100)		68 (100)	61 (89.7)	62 (91.2)	64 (95.5)	65 (97.0)	65 (97.0)
Food texture	1	100.0 ± 0.0	52.9 ± 26.5^a^	75.4 ± 27.1^b^	82.7 ± 23.4^c^	92.6 ± 21.6^d^	81.3 ± 37.3^e^
Drink consistency	1	100.0 ± 0.0	69.5 ± 32.9^a^	75.4 ± 27.1	83.8 ± 24.3^c^	90.4 ± 24.8	80.5 ± 37.6
Global health	1	33.5 ± 21.9	11.0 ± 16.4^a^	16.2 ± 18.7^b^	19.9 ± 17.0	23.5 ± 17.2^d^	29.1 ± 19.6^e^
Total score	48	4266.3 ± 232.0	1992.9 ± 1062.4^a^	2473.9 ± 962.9^b^	3169.2 ± 753.6^c^	3696.7 ± 718.3^d^	3910.8 ± 1510.4

Data are shown as mean ± standard deviation or n (%)

^a^*P* < 0.05, postoperative 2 weeks vs. preoperative 1 d

^b^*P* < 0.05, postoperative 4 weeks vs. postoperative 2 weeks

^c^*P* < 0.05, postoperative 12 weeks vs. postoperative 4 weeks

^d^*P* < 0.05, postoperative 24 weeks vs. postoperative 12 weeks

^e^*P* < 0.05, postoperative 48 weeks vs. postoperative 24 weeks

## Discussion

The aim of this study was to investigate swallowing function and swallowing-related QoL in patients with laryngeal carcinoma after partial laryngectomy. Swallowing dysfunction occurred in 72.1% of patients immediately after surgery but recovered slowly until 48 weeks, at which time 11.9% of patients were still experiencing abnormal swallowing function. The SWAL-QOL total score was negatively impacted upon by surgery but increased gradually after operation, but there was no statistical difference between SWAL-QOL total score at 48 weeks and 24 weeks postoperatively. These results revealed that in some patients, nearly a year after surgery, swallowing function and swallowing-related QoL were not restored. Therefore, more attention should be paid during postoperative nursing to improve swallowing function. For instance, patients should be trained to overcome the psychological barriers of swallowing. Because of the fear that coughing resulting from swallowing may affect wound healing, many patients do not dare eat or swallow, just keeping food in the mouth. In this case, nurses should be patient in explaining that the wound has already healed and mild coughing would not affect recovery, in order to encourage the patients to relax and swallow boldly. In addition, health care professionals should help the patients identify their suitable eating position.

After partial laryngectomy, the degree of swallowing dysfunction is directly related to the type of operation, the resection area and the larynx reconstruction method [23, 24]. In addition, the patient’s physical quality, personal tolerance and the attitude toward the disease also have indirect effects on swallowing function [25]. In this study, the water swallow test was used to evaluate swallowing function in laryngeal cancer patients after partial laryngectomy. The results showed that the incidence of dysphagia was increased by the operation but progressively decreased afterwards until 24 weeks postoperatively, with lower rates than those obtained from a dysphagia study in 38 cases of partial excision of laryngeal cricoid cartilage by Lin et al. [26], but similar to self-reported rates of dysphagia after laryngectomy internationally [24]. The reason for such difference with the above Chinese non-randomized clinical trial [26] might be that the widely used horizontal laryngectomy methods remove the structures of the hyoid body, upper thyroid cartilage and epiglottis, which play pivotal roles in the process of swallowing. The present findings demonstrated that the swallowing function in laryngeal cancer patients after partial laryngectomy is gradually improved, and the rate of dysphagia decreased. This showed that (1) although partial laryngectomy has destroyed the anatomical structure of the larynx, partial swallowing function can be compensated gradually by the patients’ postoperative self-exercise; (2) local nerve injury and muscle function of the larynx could be self-healed, recovered or compensated as time elapses; (3) 28.4% of patients still had dysphagia at 24 weeks after laryngectomy, indicating that it takes long for patients to improve swallowing function by self-exercise without medical treatment and nursing intervention. From the third day after the operation, the following was performed: (1) training of muscles and joints around the mouth (the patients were given mandibular joint, masticatory muscle and lip movement training); (2) tongue training (the patients were able to open the mouth, stick out the tongue and swing it up, down, or to left and right; (3) empty swallowing training (the patients should perform swallowing movements alternately when raising or bowing the head). The long-term impact of partial laryngectomy on dysphagia was also observed in another Chinese study [22]. Indeed, the latter authors showed that swallowing QoL remains altered more than one year after partial laryngectomy, with some patients seriously burdened by deglutition disorder, indicating that the effect of long-term dysphagia on QOL is multi-dimensional [22]. Therefore, more efforts should be made to improve swallowing function postoperatively upon partial laryngectomy in Chinese patients.

Because the larynx has important functions in swallowing, breathing and speaking [2], laryngeal cancer patients experience speaking and swallowing dysfunctions to various degrees after partial laryngectomy, which also affect the physiological, psychological and social functions of the patients [27]. In this study, we focused on swallowing-related QoL and found that SWAL-QOL total score increased gradually at 2, 4, 12, 24 and 48 weeks after laryngectomy. These results were better than those reported in patients after total laryngectomy by Zhang et al. [28] The reason may be that swallowing, breathing and speech functions of the throat are completely lost after total laryngectomy. Total laryngectomy requires the patient to carry a tube which could cause cough, expectoration, and inappropriate appearance. The patients need to learn and adapt to alternative methods of speech, all of which seriously affect their QoL. The present study also showed that SWAL-QOL total scores gradually increased with time, although there was no significant difference (*P* = 0.862) between postoperative 24 and 48 weeks. This may be explained by the following reasons. (1) The nerves and muscles could recover, and swallowing function could gradually improve after partial laryngectomy. (2) Most laryngeal cancer patients could psychologically confront difficulties such as persistent cough or expectoration with positive attitudes after medical staff education when they are aware of the likely good prognosis with high survival rate.

The results of this study highlight the need to implement methods for improving swallowing function after laryngectomy. A previous study suggested that a patient support network may help solve swallowing problems using a patient-to-patient communication model [17]. Then, a clinical study was carried out to evaluate the effectiveness and cost-utility of a guided self-help exercise program among patients treated by total laryngectomy [29]. Long-term studies involving this patient population are also needed as it has been suggested that even the patients who successfully recover swallowing after function-preserving laryngeal surgery may lose this function later; indeed, patients present with aspiration 11–15 years after partial laryngectomy and require definitive total laryngectomy [30].

This study also has some limitations. First, healthy patients were not included, and the data lack reference values. In addition, data relating to postoperative exercise and nursing intervention for patients were not collected and analyzed. Meanwhile, the quite small sample size may have introduced some biases. Larger studies with longer follow-up are needed to determine comprehensive and long-term impacts and related factors of partial laryngectomy on swallowing function and swallowing-related QoL.

## Conclusions

Partial laryngectomy is a commonly used therapy in laryngeal carcinoma to possibly maintain laryngeal function. Long-term swallowing function and swallowing-related QoL are both adversely affected in some patients even 48 weeks after surgery. This highlights the need for nursing staff to pay more attention to changes in swallowing function and swallowing-related QoL for laryngeal carcinoma patients after partial laryngectomy, and provide the necessary education and/or training to patients.

## Data Availability

The datasets used and/or analyzed in the current study are available from the corresponding author upon reasonable request.

## Abbreviations

AJCC: American Joint Committee on Cancer
QoL: Quality of life
SD: Standard deviation
SWAL-QOL: Swallow Quality-of-Life Questionnaire
